# An Opinion Paper on the Cultural Heritage of Wine Consumption in the Frame of the Mediterranean Diet: The Missing Piece of the Puzzle

**DOI:** 10.3390/nu18203374

**Published:** 2026-10-14

**Authors:** Matina Kouvari, Kalliopi Georgakouli, Rena I. Kosti

**Affiliations:** 1 Department of Nutrition and Dietetics, Nutritional Epidemiology and Communal Health Laboratory, School of Physical Education, Sports and Dietetics, University of Thessaly, 38221 Trikala, Greece; 2 Department of Nutrition and Dietetics, School of Physical Education, Sport Science and Dietetics, University of Thessaly, 42130 Trikala, Greece; kgeorgakouli@uth.gr

**Keywords:** Mediterranean diet, wine, alcohol, Mediterranean alcohol-drinking pattern, moderation, lifestyle, nutritional epidemiology, public health

## Abstract

The role of wine within the Mediterranean diet remains one of the most debated and paradoxical issues in nutritional epidemiology. Although moderate wine consumption, particularly alongside meals, has traditionally been incorporated into Mediterranean diet scores and has been associated with lower cardiovascular and all-cause mortality, alcohol is also an established carcinogen, with evidence of increased cancer risk even at relatively low levels of consumption. This apparent contradiction has renewed the debate over whether wine represents a genuine protective component of the Mediterranean diet or whether its observed associations reflect methodological and contextual factors. In this opinion paper, we critically examine emerging evidence on wine consumption, the Mediterranean dietary pattern and the Mediterranean alcohol-drinking pattern, with particular attention to the limitations of observational alcohol epidemiology, including abstainer-reference bias, reverse causation, exposure misclassification and the divergence between observational and Mendelian randomization evidence. Additionally, drawing on the concept of the traditional Mediterranean lifestyle, we further propose that moderation and synergy between lifestyle components may be more informative than the isolated attribution of health effects to individual foods or beverages. Thus, instead of searching for a “hidden virtue” in wine itself, the more relevant hypothesis may be that of a “hidden context”, whereby the health associations attributed to moderate wine consumption are inseparable from the broader Mediterranean lifestyle in which it is embedded.

## 1. Introduction

For more than two decades, the Mediterranean diet has held a privileged place in nutritional science, repeatedly confirmed in cohort studies and landmark clinical trials such as the Lyon Diet Heart study and the PREDIMED trial to be associated with lower cardiovascular and all-cause mortality risk [1,2,3]. Olive oil, vegetables, legumes, nuts, and fish are usually credited as the pattern’s core protective components [4]. Notably, most of the validated Mediterranean diet indexes also assign a favorable weighting to moderate alcohol intake or specifically to moderate wine intake, consumed with meals, recognized as beneficial only within a narrowly defined range of consumption [5]. About two years ago, in a widely discussed narrative review, Martínez-González [6] directly raised the hypothesis of whether wine should be removed from the Mediterranean dietary pattern, noting that removing the alcohol item from Mediterranean diet scores has been shown to attenuate the pattern’s inverse association with all-cause mortality [7,8,9]. Hence, currently, the primary question is not whether wine correlates with the benefits attributed to the Mediterranean diet but whether this correlation reflects something causal, something cultural and/or something that nutrition epidemiology has been repeatedly measuring incorrectly.

Recently, an analysis in two large Spanish cohorts—the PREDIMED clinical trial and the SUN study—was designed specifically to separate the contribution of wine from the rest of the Mediterranean dietary pattern, examining whether benefits attributed to “the Mediterranean diet” are related to the extent to which wine is part of it [10]. In the PREDIMED analysis, participants at increased cardiometabolic risk who closely followed the Mediterranean diet but without occasional wine consumption saw a 23% reduction in total mortality, while those who also drank wine moderately—seven to twenty-one glasses per week—saw a 33% reduction. In addition to this, within the same study, a nested case–cohort analysis showed that light-to-moderate wine consumption, measured through an objective biomarker (tartaric acid), was prospectively associated with lower cardiovascular disease incidence [11]. The aforementioned ten-point gap along with the positive results generated after an objective measurement of red wine consumption is the empirical heart of the hypothesis, where wine probably acts beyond synergistically with the rest components of the Mediterranean diet, being an indispensable part of Mediterranean culture that cannot be disregarded [12].

One of the strongest claims for the health benefits of red wine, particularly for cardiac health, emerged from the “French paradox”—the observation of low heart disease rates despite a high-fat diet, originally credited to red wine and its resveratrol content [13]. While this specific explanation has not been adequately confirmed, a substantial body of prospective epidemiological evidence has supported an inverse association between low-to-moderate wine consumption and all-cause mortality as well as a nonlinear association between wine consumption and cardiovascular disease incidence [7,8,9,14]. Such findings are reinforced by the approach used in the Mediterranean Alcohol-Drinking Pattern (MADP) index—distinct from raw quantity—which captures how Mediterranean populations traditionally drink; they favor wine over spirits in moderation, with food, and in the context of communal dining, alternating it with water and spreading consumption across the week rather than concentrated into binges [15]. The results from two southern Italian cohorts followed from 2006 to 2022 found that the effects of alcohol on mortality vary, depending on alcohol consumption patterns and dietary context. Specifically, drinking alcohol in the Mediterranean way—in moderation, usually wine, and with meals—while also following the Mediterranean diet appears to reduce some of the harmful effects attributed to alcohol compared to other drinking patterns [15]. Indeed, the implication that many researchers have drawn is that it may not be the alcoholic beverage per se that drives any apparent benefit but the entire constellation of how, when, and alongside what it is consumed, which is difficult to separate from socioeconomic and lifestyle confounders [16].

On the other hand, the International Agency for Research on Cancer (IARC) has taken the same position for years: alcohol is a Group 1 carcinogen, and, according to the latest evidence summary of the IARC, even a “moderate” drinking pattern consisting of under two drinks a day is related to increased cancer risk [17,18]. This has produced a noticeable shift in how alcohol is discussed in public health communication, specifically focusing on the carcinogenic effect of ethanol, despite any protective or neutral signal for light drinking [19]. Regarding this issue, in 2022, an estimated 146,194 cancer cases in the EU were attributed to alcohol [20]. Increases in the relative risk for alcohol-related cancers, especially breast cancer, appear even at low intake; breast cancer is common, so a small relative increase translates into a real number of cases [21]. Hence, the most prominent current public health messaging focuses specifically on cancer endpoints [22]. The biological mechanisms that connect ethanol with cancer (principally acetaldehyde-mediated DNA damage) are the least contested in the literature, and public awareness of the alcohol–cancer link remains low, maximizing the communicative yield of this approach [20,23]. One widely cited absolute-risk comparison found that the consumption of a single bottle of wine a week carries a lifetime cancer risk roughly equivalent to ten cigarettes a week for women and five for men, an association assessed precisely to make the risk tangible by comparing it with a familiar and socially recognized harmful behavior [24]. As a strategy for raising awareness, this approach is defensible, yet it is different from estimating the net health effect, which requires weighing cancer risk against competing risks such as cardiovascular diseases. For instance, in the aforementioned study with the direct comparison on the cancer effect size of tobacco use and wine intake, wine was treated as equivalent to ethanol. In addition to this, as in many similar observational studies, the consumption pattern or overall lifestyle attitudes were systematically ignored, oversimplifying the complexity of the topic [24]. Overall, in the present manuscript, we raise the hypothesis that the health associations attributed to moderate wine consumption are inseparable from the broader Mediterranean lifestyle in which it is embedded. The scope of this work is to critically discuss the current evidence in the field.

## 2. The Measurement Error: A Challenge Related to Both Positive and Inverse Associations

Before any causal claim about wine can be accepted, it has to overcome a methodological gap that alcohol epidemiology has historically failed to. The most consequential issue is the “sick-quitter” or “abstainer-reference” problem. Many studies compare moderate drinkers against a reference group of “non-drinkers” that includes people who quit drinking because they were already ill [25,26]. This bias does not run in only one direction. Simply excluding former drinkers, or treating them as a separate group, results in residual bias, since their poor health outcomes arguably belong with the drinking groups that reflect their lifetime consumption, not with the abstainers. A second issue is reverse causation operating earlier than quitting. People who reduce their drinking because of emerging symptoms are assigned to the “moderate” category before the official diagnosis of their underlying illness. A proof study of alcohol and ischemic heart disease published in *Nature Communications* in 2024 laid out both problems together, noting that failing to separate lifetime abstainers from former drinkers who may have quit due to the development of preclinical symptoms can introduce reverse causation bias and that estimates can additionally be distorted by measurement error from inaccurate self-reports, fluctuating consumption over time, or socially desirable underreporting [27]. Indeed, alcohol intake is almost universally underreported in self-administered dietary questionnaires, often by 40–60% relative to actual sales data [28]. Last, we have to consider potential socioeconomic confounding effects. For instance, there is evidence in the literature that adults who drink alcohol are markedly different from adults who do not drink alcohol in terms of socioeconomic background. Thereby, concerns remain that the lower mortality observed in the context of moderate drinking may be attributed primarily to the favorable risk factor profile seen in moderate drinkers [29].

Mendelian randomization, which uses genetic variants associated with alcohol metabolism as a proxy for lifetime exposure, has generally not reproduced the protective J-shaped association seen in observational cohorts. A recent work examined alcohol consumption and the risk of all-cause and cause-specific mortality through both linear and nonlinear Mendelian randomization, revealing monotonically increasing risk rather than a protective dip at low intake [30]. Even if the Mendelian randomization methodology has important limitations, this deviation between observational and genetic instrument evidence is arguably an important reason to treat any wine-specific mortality with caution, questioning whether the observational signal is a matter of confounding or a matter of biology.

In response to such contradictory claims, the UNATI trial in Spain is randomizing roughly 10,000 regular drinkers to either abstention or continued moderate intake, following them for four years with biomarker-verified compliance, including hair ethyl glucuronide testing, an objective marker of alcohol exposure that sidesteps self-report bias entirely. Its primary endpoint is a composite of mortality, cardiovascular events, cancer, cirrhosis, diabetes, depression, dementia, injury, and serious infection, deliberately built to let cancer and cardiovascular signals be weighed against each other rather than reported in isolation [31]. This trial reflects the current genuine state of equipoise in the scientific community.

## 3. Wine, Health and Culture: From a “Hidden Virtue” to a “Hidden Context”

Considering the evidence discussed above, the question may not be whether wine possesses a “hidden virtue” but whether its contribution to the Mediterranean dietary pattern is attributed to the context in which wine is traditionally consumed. This distinction is important because the MADP already indicates that wine cannot be meaningfully interpreted independently of the consumption pattern. The type of beverage, consumption in moderation, consumption during meals, the distribution of intake across the week and the social setting are all characteristics of the same behavioral pattern. Thus, in epidemiology, moderate wine consumption may represent more than exposure to ethanol or wine-derived compounds.

This interpretation also offers a more coherent way of understanding why apparently contradictory findings can coexist, indicating that the exposure being studied is embedded within a broader behavioral configuration. In particular, the relevant unit of interpretation may be the pattern rather than the beverage. This is consistent with the broader concept of the Mediterranean diet itself. The health effects of the Mediterranean dietary pattern have traditionally been discussed in terms of isolated foods—olive oil, vegetables, legumes, nuts, fish and other characteristic components. However, in real life, these components are not consumed independently. Their potential effects emerge within a pattern characterized by particular combinations, quantities and frequencies of consumption. A similar principle could apply to wine.

Our recent review of the traditional Mediterranean lifestyle provides a useful framework for interpreting this issue [32]. Rather than considering the Mediterranean diet as a list of foods with additive effects, we examined it as part of a wider lifestyle in which moderation and synergy between its components are central principles. From this perspective, moderation is not simply the amount of a particular food or beverage that falls below an epidemiological threshold. It is a characteristic of the overall behavioral pattern. The value of the Mediterranean lifestyle may therefore derive from the way its dietary, social and behavioral elements reinforce one another rather than from the isolated effect of any single component. This distinction is particularly relevant to wine [6]. Regarding this issue, removing wine from a Mediterranean diet score may weaken the score’s statistical association with health outcomes without necessarily demonstrating that wine itself is responsible for the association. Wine may partly act as a marker of adherence to the wider behavioral and cultural pattern within which the Mediterranean diet operates.

Such an interpretation does not dismiss the possibility that wine-specific elements may have biological effects. The polyphenol content of wine varies according to grape variety, production practices and other characteristics, and experimental evidence has suggested the potential biological effects of specific wine-derived compounds [33,34,35,36]. Nevertheless, demonstrating that a compound can exert a biological effect is not equivalent to demonstrating that wine consumption, at the population level, produces a net health benefit. Similarly, the existence of a favorable association between wine and health in a particular observational context does not establish that wine is a necessary component of the Mediterranean dietary pattern. The distinction between biological potential and demonstrated health benefit is therefore essential.

The concept of synergy may help explain why this distinction matters [32]. If the health effects of the Mediterranean pattern arise partly through interactions among its components, then attempts to isolate one component may provide an incomplete picture. A food, beverage or behavior can be associated with the pattern without being independently responsible for its overall effects. Wine is a good example of this matter since its potential benefits are simultaneously challenged by well-established harms associated with alcohol exposure.

This broader interpretation also changes the meaning of moderate wine consumption. Instead of viewing moderation solely as a range of ethanol intake, it may be more appropriate to consider it one manifestation of a wider Mediterranean principle [31]. In our recent review, moderation was discussed in relation to the interaction between the different dimensions of the traditional Mediterranean lifestyle, emphasizing that its health potential cannot be fully captured by isolated components [32]. Applied to the current debate, this suggests that the relevant characteristic may not be that Mediterranean populations drank wine, but that wine intake occurred within a lifestyle in which excess was traditionally discouraged and food, social interaction and everyday practices were closely interconnected [32]. However, the lifestyle in the current era within the Mediterranean region with all the elements of Westernization is something that needs to be taken into consideration [37]. Hence, maybe it is not the wine itself but the whole context of consumption that has changed.

This perspective has an important implication for the interpretation of the current evidence. The fact that wine contributes to the predictive performance of Mediterranean diet scores should be neither ignored nor directly translated into a recommendation to consume wine. The same epidemiological finding can be interpreted in two fundamentally different ways: either wine contributes directly to the health effect of the Mediterranean diet, or wine consumption captures, at least partly, adherence to a broader pattern in which moderation and synergy are fundamental characteristics. As previously mentioned, existing observational evidence cannot fully distinguish between these possibilities.

The forthcoming results of UNATI [6] will therefore be important not only because they may clarify the health effects of moderate alcohol intake but also because they may help define the limits of what can reasonably be inferred from observational Mediterranean diet research [31]. In the meantime, the most defensible interpretation may be to move to the recognition of the context in which wine is consumed: the possibility that the apparent role of wine reflects the interaction between the beverage, the circumstances of its consumption and the broader Mediterranean lifestyle in which it is embedded.

## 4. Conclusions

The current evidence does not point in a single direction. First, within Mediterranean diet scores, removing the wine component measurably weakens the pattern’s association with lower mortality, and this finding has now been replicated across different cohorts. Second, this statistical contribution cannot yet be read as proof that wine can be protective or harmful because of various methodological gaps. Third, the literature systematically treats alcohol consumption in terms of ethanol equivalents; however, wine—as all alcoholic beverages—is not simply ethanol. Production practices vary substantially across countries and regions, yielding products that differ not only in ethanol content but, more importantly, in their profile of bioactive compounds [31]. Fourth, the type of alcoholic beverage matters; for instance, red wine—primarily due to its polyphenol content—has been suggested to enhance hormesis—a biphasic adaptive response in which low doses of even potentially harmful substances can provide benefits for survival, longevity and stress resistance [38,39]. However, more mechanistic proofs in the field are demanded in terms of at which quantity such benefits can be exerted. Last, the “hidden virtue” framing may itself be somehow misleading. What the MADP literature suggests is that any potential benefits of wine may be inseparable from the context in which it is consumed—how, when, and alongside what foods—as well as from the broader socioeconomic, commercial and behavioral profile, rather than from the independent health effects of ethanol or polyphenols per se. Additionally, MADPs may reflect broader lifestyle and socioeconomic factors rather than independent protective effects. Such arguments extend to the Mediterranean dietary pattern as a whole. Until the results from the UNATI trial [6] and comparable randomized evidence are reported, the more balanced position is to treat wine’s place in the Mediterranean diet as an open, well-motivated hypothesis rather than an established protective or harmful factor, acknowledging in parallel the cultural and culinary importance of wine as part of a convivial dietary pattern.

## Data Availability

No new data were created or analyzed in this study. Data sharing is not applicable to this article.
